# Cardiovascular Safety of Hormonal Contraception: Method-Specific Risks and Clinical Implications

**DOI:** 10.3390/medsci14020201

**Published:** 2026-04-16

**Authors:** Iga Waluszewska, Antoni Borowiec, Alicja Paciorek, Letycja Musz, Wioletta Szczurek-Wasilewicz

**Affiliations:** 1 Students’ Scientific Society, Department of Pharmacology, Faculty of Medicine, University of Opole, 45-052 Opole, Poland; igawaluszewska@gmail.com (I.W.); antoni.borowiec2@gmail.com (A.B.); alicjapaciorek6@outlook.com (A.P.); muszletycja@gmail.com (L.M.); 2 Department of Pharmacology, Faculty of Medicine, University of Opole, 45-052 Opole, Poland

**Keywords:** hormonal contraception, risk stratification, cardiovascular risk, prothrombotic risk factors, thromboembolism, myocardial infarction, ischemic stroke

## Abstract

Hormonal contraception is used by hundreds of millions of women worldwide and remains one of the most effective reversible methods of pregnancy prevention. Cardiovascular (CV) safety concerns, particularly venous thromboembolism (VTE), ischemic stroke, myocardial infarction, and blood pressure elevation, are important considerations when choosing forms of contraception. Estrogen-containing combined hormonal contraceptives (CHCs) increase the relative risk of VTE; however, among healthy young nonsmokers, absolute event rates remain low. Risk is strongly modified by estrogen dose, progestin type, route of administration, and individual factors such as age, smoking, migraine with aura, hypertension, obesity, inherited thrombophilia, the postpartum period, and concomitant prothrombotic medications. Progestin-only contraceptives and levonorgestrel-releasing intrauterine systems (LNG-IUSs) generally show a more favorable thrombotic profile and are preferred options for women with contraindications for estrogen. This review summarizes current evidence on the method-specific CV risks of hormonal contraception, highlights the mechanisms underlying these effects, and provides practical guidance for clinical decision-making.

## 1. Introduction

Hormonal contraception (HC) is widely used worldwide. Types include oral pills and non-daily delivery systems such as vaginal rings, transdermal patches, injectables, implants, and hormonal intrauterine devices (IUDs). HC primarily prevents pregnancy by suppressing ovulation and altering the endometrium and cervical mucus, thereby reducing the likelihood of fertilization and implantation [1,2,3,4]. HC use is widespread globally, and its cardiovascular (CV) safety is therefore clinically relevant in routine practice [5].

Beyond contraception, selected HC options provide clinically relevant non-contraceptive benefits, including regulating menstrual bleeding, reducing dysmenorrhea, treating endometriosis, ameliorating acne, and managing polycystic ovary syndrome (PCOS) [2,3,4]. Moreover, certain HC formulations have been developed for emergency contraception after unprotected sexual intercourse [4].

Despite their high efficacy and non-contraceptive benefits, some hormonal methods are associated with systemic physiological changes. For many patients, concerns regarding adverse effects, particularly CV safety, remain a significant barrier to use [1,6]. Clinical evidence indicates that HC can influence the CV system, potentially increasing the risk of thromboembolic events, including venous thromboembolism (VTE), ischemic stroke, and myocardial infarction (MI). Importantly, these risks are method-specific and influenced by estrogen dose, progestin type, and route of administration as well as individual baseline risk [5,6,7,8]. Although absolute risks are generally low for young, healthy women, risk varies by method and increases in the presence of pre-existing CV risk factors and selected forms of co-exposure or drug interactions. Both non-modifiable factors (e.g., age and genetic predisposition) and modifiable factors related to lifestyle should be carefully considered when selecting an appropriate contraceptive regimen [7,8]. This review summarizes contemporary evidence on the CV effects and clinical outcomes associated with different HC methods, highlighting method-specific risk profiles and implications for clinical practice and individualized counseling.

Despite the introduction of lower-dose estrogen formulations, CV safety remains a clinically relevant concern [9,10]. Even modern combined hormonal contraceptives (CHCs) retain estrogen-related hepatic effects that promote a procoagulant state, and the associated risk of thrombosis persists, particularly in the presence of additional patient-level risk factors such as smoking, obesity, or hypertension [11,12,13,14,15]. Importantly, increases in relative risk may translate into clinically meaningful absolute risk in real-world settings [9,10]. Furthermore, observational data suggest that the risk of thrombosis varies across formulations and routes of administration, indicating that reductions in estrogen dose alone do not fully eliminate CV risk [9,10,16,17].

The increasing use of long-acting reversible contraception (LARC) offers highly effective, low-maintenance options for women seeking reliable birth control [11]. LARC methods, particularly those without systemic estrogen, are generally associated with a lower risk of VTE compared with CHCs [18]. However, certain systemic progestin-containing LARC methods may still confer a small but measurable risk of thrombosis in women with additional risk factors, such as obesity or thrombophilia [10]. Understanding these nuances is important for counseling women about CV safety when selecting contraceptive methods [10,11,18].

## 2. Methods—Search Strategy

This narrative review was based on targeted PubMed searches conducted using combinations of keywords related to HC and CV safety, including “combined hormonal contraception”, “progestin-only”, “venous thromboembolism”, “stroke”, “myocardial infarction”, “hypertension”, “patch”, “vaginal ring”, and “levonorgestrel intrauterine system”. The cited literature covered the period from 1993 to 2025, with particular emphasis on contemporary evidence from 2000 onward.

We prioritized high-quality evidence, including systematic reviews and meta-analyses, large cohort and registry-based studies, randomized or pharmacodynamic studies where relevant, and major clinical guidelines and expert recommendations. Older landmark publications were included where necessary to provide historical or mechanistic context.

Publications in languages other than English were excluded. Case reports, small case series, non-human studies, and publications not directly relevant to CV outcomes or contraceptive safety were also excluded. When conflicting evidence was identified, greater weight was given to larger, more recent studies and guideline-based recommendations.

### 2.1. Hormonal Contraception Methods

HC can be categorized by hormone composition—distinguishing combined estrogen and progestin methods from progestin-only methods—and route and dosing frequency, including daily oral pills and non-daily delivery systems [2,19]. The route of administration influences pharmacokinetics and systemic exposure, which may translate into differences in both efficacy and adverse-effect profiles [2,19,20].

Combined oral contraceptives (COCs) most commonly contain ethinyl estradiol (EE) (typically 20–35 µg) combined with a progestin. Newer COCs may contain natural estrogens (e.g., estradiol valerate) or estetrol (E4), which available data suggest may have less pronounced hepatic, hemostatic, and metabolic effects compared with EE-containing products, although clinical outcome data remain limited [2]. Progestin-only pills (POPs), injectable depot medroxyprogesterone acetate (DMPA), the etonogestrel implant, and levonorgestrel-releasing intrauterine systems (LNG-IUSs) are preferred options when estrogen is contraindicated [2,15].

The combined vaginal ring (e.g., etonogestrel/EE) provides relatively stable systemic hormone levels over 3 weeks of use, followed by a 1-week hormone-free interval [21]. The combined transdermal patch (norelgestromin/EE) is typically changed weekly for three consecutive weeks followed by a patch-free week. Pharmacokinetic comparisons indicate that overall EE exposure is higher with the patch than with many oral formulations, a finding that is clinically relevant when considering the risk of thrombosis [20,22]. In addition, patch effectiveness may be reduced in some users with higher body weight (e.g., ≥90 kg) [23]. The patch can be applied to the buttocks, abdomen, upper outer arm, or upper torso excluding the breast area. Pharmacokinetic data suggest hormone exposure is approximately 20 percent lower when the patch is placed on the abdomen compared with placement on other sites, although the mean concentrations generally remain within therapeutic ranges [24].

The 52 mg LNG-IUS provides LARC with predominantly local uterine effects and very low systemic levonorgestrel concentrations. Extension studies indicate that 52 mg LNG-IUS devices provide contraceptive efficacy and safety over 8 years of use [25,26].

Emergency contraception (EC) options include levonorgestrel and ulipristal acetate oral regimens and, when feasible, copper IUD insertion (which subsequently provides ongoing long-acting contraception). The available evidence does not suggest single-dose EC regimens pose an increased risk of VTE; however, EC does not address ongoing contraceptive needs and should prompt follow-up counseling on an appropriate long-term method [17,27].

The key characteristics of commonly used HC methods and their main CV considerations are summarized in Table 1.

### 2.2. Hormonal Contraceptive Functions and Mechanisms

The menstrual cycle is regulated by the hypothalamic–pituitary–ovarian (HPO) axis. Pulsatile gonadotropin-releasing hormone (GnRH) from the hypothalamus stimulates the anterior pituitary gland to secrete luteinizing hormone (LH) and follicle-stimulating hormone (FSH), which coordinate follicular development and ovarian steroidogenesis, including the production of estradiol (E2) and progesterone. Physiologically, variation in GnRH pulse frequency contributes to the differential regulation of LH and FSH synthesis and underpins the transition from tonic pulsatile secretion to the preovulatory gonadotropin surge [28,29].

Most systemic forms of HC exert their primary effect through negative feedback on the HPO axis. Progestins are the main drivers of ovulation inhibition, functioning by suppressing gonadotropin secretion—particularly LH—thereby preventing the mid-cycle LH surge; concomitantly, the absence of normal follicular maturation limits the rise in endogenous E2 levels that is required for positive feedback [30,31,32]. Importantly, the relative contribution of central ovulation suppression versus peripheral mechanisms differs across preparations and doses, a phenomenon that is especially relevant for progestin-only methods [30,33]. Additional contraceptive mechanisms include thickening of cervical mucus, endometrial thinning/atrophy, and alteration of tubal motility [30,31].

In CHCs, the estrogen component further suppresses follicular recruitment by reducing FSH secretion and stabilizes the endometrium, thus potentially reducing unscheduled bleeding [30,31]. Across systemic methods, CHCs generally provide more consistent ovulation suppression and better cycle control than traditional low-dose POPs. However, the inhibition of ovulation via POCs is formulation-dependent; the extent is high with the desogestrel (75 μg) POP and maintained with the drospirenone (4 mg) (24/4 regimen) POP [33,34,35].

In contrast, LNG-IUSs function predominantly via local mechanisms (thickening of cervical mucus and endometrial suppression) with limited systemic exposure [31,36]. This predominantly local mode of action is explained by low circulating levonorgestrel levels, which are generally insufficient for reliable ovulation inhibition; therefore, most women using an LNG-IUS continue to ovulate regularly [9].

### 2.3. Determinants of the Risk of Thrombosis Across CHC and Progestin-Only Contraception

Beyond contraceptive mechanisms, the CV safety of HC is largely formulation-dependent and driven by systemic estrogen exposure and hepatic effects [11,37,38]. Accordingly, estrogen-containing CHCs carry the primary thrombotic signal, whereas progestin-only methods generally display a more favorable thrombotic risk profile [11,37,39,40]. From a CV perspective, estrogen type and dose are key determinants of the risk of thrombosis, while the progestin component can modulate hemostatic effects and cardiometabolic profiles [11,37,38].

Although both classes ultimately achieve contraception through progestin-mediated effects, their CV safety profiles diverge primarily because of the estrogen component and formulation-specific pharmacokinetics. Key determinants include estrogen type and dose (classically EE), progestin type (with variable androgenic/anti-androgenic and anti-mineralocorticoid properties), and the route of administration, which influences systemic exposure and, for oral preparations, hepatic first-pass effects and hepatic protein synthesis [11,37,38,39,40,41,42].

EE has potent hepatic effects that increase synthesis of procoagulant factors and alter anticoagulant pathways, shifting hemostasis toward a prothrombotic phenotype [38]. These effects are dose-dependent and may be influenced by systemic exposure related to the route of administration [37,41,42]. Large registry datasets show that EE-containing CHCs pose a greater risk of VTE, which varies by formulation and route (including non-oral systems such as transdermal patches and vaginal rings) [37,41].

In absolute terms, the baseline risk of VTE in reproductive-age women not using HC is low, estimated at approximately 1–3 cases per 10,000 woman-years [37,41]. The use of CHCs increases this risk to approximately 5–12 cases per 10,000 woman-years, depending on the formulation and population characteristics [11,37,43]. For comparison, the risk is higher during pregnancy and increases substantially in the postpartum period, reaching up to 40–65 cases per 10,000 woman-years [11,44]. These comparisons are particularly relevant in clinical counseling, as they help contextualize relative risk increases associated with hormonal contraception.

Importantly, the risk of VTE is not uniform across CHCs. Epidemiologic evidence indicates that formulations with similar estrogen doses may confer different risks of VTE depending on the accompanying progestin, supporting the notion that the progestin component is not biologically neutral [37,43].

Most of the available evidence on the risk of VTE associated with HC is derived from observational cohort and registry-based studies, as randomized trials are not feasible for rare thrombotic outcomes. While these studies provide valuable real-world data, they are subject to limitations such as residual confounding, confounding via indication, and prescribing bias. Therefore, findings should be interpreted with caution, particularly when comparing different contraceptive formulations [11,37].

Newer estrogenic components are being explored to potentially reduce hemostatic perturbations. In observational data, COCs containing natural estrogens, mainly E2 or estradiol valerate, have been associated with a lower risk of VTE than EE-containing COCs, although confounding and formulation selection may influence these estimates. A recent large meta-analysis indicated that natural-estrogen-based COCs pose a 33% lower risk of VTE than synthetic-estrogen-based formulations [45]. E4 is a newer estrogen that has shown a comparatively favorable hemostatic profile in short-term pharmacodynamic studies, but long-term comparative clinical outcome data remain limited [10,46,47].

Arterial thrombotic events (ischemic stroke and MI) appear to be strongly related to estrogen exposure and have mainly been associated with higher-dose CHCs. However, contemporary low-dose formulations are associated with lower absolute risks, which remain clinically relevant among individuals with additional risk factors such as smoking, hypertension (HTN), and migraine with aura. Overall, the risk of ischemic stroke and MI appears to be primarily determined by estrogen dose, while differences across progestin types are less consistent than those observed for VTE [42,43].

In contrast, most POC methods (POPs, implants, and LNG-IUS) appear to carry little or no increased risk of VTE compared with non-use, consistent with their estrogen-free profiles [11,40]. DMPA may be an exception, as noted in some datasets and systematic reviews, with indications of increased risk of VTE in selected populations, warranting individualized assessment [39,40]. Accordingly, when the risk of thromboembolism or CV events is elevated, estrogen-containing CHCs are often avoided, and progestin-only methods are preferred alternatives [11,40]. Among POCs, newer POP formulations designed for more reliable ovulation suppression (e.g., desogestrel (75 μg) and drospirenone (4 mg)) may improve bleeding patterns while maintaining estrogen-free CV advantages [34,35].

Importantly, the risk of thrombosis and CV risk are dynamic and may evolve over time. They may change with age, postpartum status, new-onset HTN or migraine with aura, initiation or resumption of smoking, weight gain/obesity, and concomitant use of medications associated with an increased risk of VTE (e.g., nonsteroidal anti-inflammatory drugs (NSAIDs)) [8,41]. Therefore, medical eligibility and thrombotic/CV risk assessment should be revisited periodically and whenever new risk factors emerge rather than treated as a one-time screening step upon initiation of contraceptive use [41,48].

### 2.4. Potential Effects of Hormonal Contraception on Cardiovascular and Cardiometabolic Risk Factors

#### 2.4.1. Lipid and Metabolic Profiles

HC exerts generally modest but formulation-dependent effects on lipid profiles and metabolic parameters. The estrogen component tends to increase triglyceride levels and may increase levels of high-density lipoprotein (HDL) cholesterol, whereas an increase in the quantity of androgenic progestins can attenuate HDL increases and may unfavorably influence low-density lipoprotein (LDL) cholesterol. In turn, anti-androgenic or non-androgenic progestins are more frequently associated with a comparatively favorable lipid profile, although the clinical significance of short-term lipid changes for long-term CV outcomes in young women remains uncertain [3,49]. In a systematic review and meta-analysis of adult premenopausal women, oral contraceptive use was associated with consistent increases in levels of triglycerides and HDL cholesterol across multiple formulations, whereas LDL cholesterol changes varied substantially by progestin type. In the same analysis, pooled effects on body mass index (BMI) and glycemic/insulin indices (fasting glucose, fasting insulin, and homeostatic model assessment of insulin resistance (HOMA-IR)) were generally small and often not statistically significant [49]. Overall, these findings suggest that the metabolic changes brought about by oral contraceptives are usually modest in healthy users but may warrant closer follow-ups for women with baseline cardiometabolic risk factors (e.g., dyslipidemia, obesity, insulin resistance, or PCOS) [3,49]). In specific populations, such as women with PCOS, certain CHC formulations (e.g., drospirenone-containing COCs) may improve selected metabolic and hormonal parameters compared with other progestins, but formulation choice should be individualized because PCOS itself confers a cardiometabolic risk [50]. Moreover, the evidence regarding PCOS is more heterogeneous and largely derived from observational studies. A systematic review and meta-analysis reported that COC use may be associated with adverse changes in insulin resistance and dyslipidemia in women with PCOS, although confounding (particularly by BMI) and differences in formulation and duration limit causal inference [16]. Consistently, another meta-analysis focusing on metabolic profiles among women with PCOS found that OC use was associated with increases in several lipid fractions (including triglycerides and LDL cholesterol), while effects on bodyweight and glucose parameters were generally small [51]. These findings call for lipid monitoring in higher-risk PCOS phenotypes rather than assuming there is metabolic neutrality across formulations [16,51].

#### 2.4.2. Blood Pressure and Fluid Retention

Beyond lipids and glycemic indices, CHCs may influence blood pressure (BP) and fluid balance. Average increases in BP are usually small, but a susceptible subgroup can develop clinically relevant HTN. This effect appears to be driven mainly by the estrogen component through hepatic upregulation of angiotensinogen and activation of the renin–angiotensin–aldosterone system (RAAS) [52,53]. BP should therefore be measured before CHC initiation and reassessed during follow-ups, and estrogen-containing CHCs are generally avoided in women with HTN according to medical eligibility criteria and clinical guidance [48,53]. Estrogen-containing regimens may also promote sodium and water retention, contributing to bloating, edema, and perceived weight gain in some users, potentially reflecting fluid shifts rather than increased fat mass. Drospirenone has antimineralocorticoid activity and may partially counterbalance estrogen-related fluid retention. Additionally, clinical studies have reported reductions in total and extracellular body water with drospirenone-containing COCs [54,55,56]. In contrast, progestin-only methods show no consistent association with increased BP and are generally preferred when HTN or fluid-sensitive states are a concern [12,57].

#### 2.4.3. Vascular Effects of Estrogens and Endothelial Function

Estrogen acts on both vascular smooth muscle and endothelial cells and may promote vasodilation and anti-atherogenic signaling through enhancing nitric oxide (NO) bioavailability, antioxidant actions, and modulating vasoconstrictor pathways [58]. Experimental and clinical data from menopausal hormone therapy support these vasodilatory mechanisms, but their clinical relevance in contraceptive users remains less clear since contraceptive estrogens differ in type and dose, are used in younger populations, and involve different routes of administration and various accompanying progestins [59,60]. Accordingly, while estrogen-related endothelial effects provide biological plausibility for favorable vascular signaling, they should not be interpreted as evidence of CV protection via CHCs, particularly given the dominant thrombotic signal driven by systemic estrogen exposure in susceptible women [42,60].

From a pathophysiological standpoint, E2 can increase NO bioavailability via both genomic and non-genomic pathways, including upregulation and activation of endothelial NO synthase (eNOS) and attenuation of NO degradation through reducing oxidative stress [58,59]. In CHC users, the concurrent progestin may further shape vascular signaling through its receptor activity and cardiometabolic properties, thereby modulating endothelial responses. Progestins differ in their androgenic/anti-androgenic and anti-mineralocorticoid activities and in metabolic effects, which may influence vascular reactivity, particularly in women with a baseline cardiometabolic risk [59,60].

#### 2.4.4. Hormonal Contraception and Inflammatory Biomarkers

HC, particularly combined methods, has been associated with higher circulating levels of markers commonly used as surrogates for low-grade inflammation, most consistently C-reactive protein (CRP) in observational studies [61,62]. In a randomized study comparing oral pills, transdermal patches, and vaginal rings among healthy young women, CRP levels increased across all routes of administration, and levels of pentraxin-3 (PTX-3) also increased in selected groups [63]. At the same time, controlled data suggest that the CRP rise observed with COC use may occur without a broader systemic inflammatory response, supporting the interpretation that, at least in part, it may reflect estrogen-related hepatic acute-phase protein (APP) synthesis rather than generalized inflammation [64]. Because high-sensitivity CRP (hs-CRP) is an established marker associated with future CV events in women, isolated elevations during CHC use should be interpreted cautiously and integrated with the overall thrombotic and cardiometabolic risk profile rather than treated as proof of “vascular protection” or, conversely, as evidence of clinically relevant inflammation in every user. Whether these biomarker changes translate into higher long-term CV risk in otherwise healthy young women remains uncertain; however, they may be more relevant when multiple cardiometabolic risk factors are present [65].

#### 2.4.5. Key Cardiovascular Risk Factors in Contraceptive Eligibility

Although the absolute CV risk in most reproductive-aged women is low, CHCs may increase the risk of thrombosis through estrogen-related procoagulant changes. Therefore, eligibility assessments should systematically include baseline CV and cardiometabolic risk factors. The U.S. Medical Eligibility Criteria for Contraceptive Use (US MEC) 2024 provides a standardized framework for method selection by grading the safety of CHCs and other methods with respect to common conditions (e.g., smoking, HTN, diabetes, obesity, and migraines). In practical terms, US MEC categories 3–4 generally indicate that estrogen-containing contraception should be avoided, while non-estrogen alternatives (LARC/progestin-only methods) are preferrable. Risk factors differ in the predominant events of concern: some primarily amplify arterial events (MI and ischemic stroke), whereas others predominantly increase the risk of VTE, and “risk stacking” (the presence of multiple atherosclerotic risk factors) often shifts CHCs toward categories where estrogen-containing methods should generally be avoided [48]. Table 2 summarizes the major CV risk factors relevant to contraceptive choice.

Multiple risk factors for atherosclerotic CV disease, e.g., older age plus smoking, diabetes, HTN, or dyslipidemia, usually place CHCs in category 3 or 4, so estrogen-containing methods should generally be avoided, and LARC or progestin-only methods should be chosen instead.

The meanings of US MEC categories are given below:

Cat. 1: No restriction;

Cat. 2: Advantages generally outweigh risks;

Cat. 3: Risks usually outweigh advantages (generally, avoid CHCs; consider alternatives);

Cat. 4: Unacceptable health risk (contraindicated) [71,72,73].

#### 2.4.6. Contraceptive Options for Women with Established Cardiovascular Disease

For women with established CV conditions, such as congenital heart disease (CHD), a history of MI or ischemic stroke, and pulmonary hypertension, the choice of contraception is strictly guided by the risk of exacerbating the underlying disease or triggering a secondary thrombotic event. According to the European Society of Cardiology (ESC) guidelines on CVD prevention and the WHO MEC, CHCs are generally contraindicated (MEC 4) for these high-risk populations due to their prothrombotic hepatic effects [71,72,73].

In patients with CHD or mechanical heart valves requiring anticoagulation, the risk of heavy menstrual bleeding must also be managed. For these women, LNG-IUSs are preferred options (MEC 2), as they provide high contraceptive efficacy with a favorable CV safety profile and localized or minimal systemic hormonal impact. For women with pulmonary arterial hypertension, LARC methods are strongly recommended, while estrogen must still be strictly avoided to prevent further pulmonary vascular compromise [71,72,73].

### 2.5. Practical Clinical Approach and Counseling

Building upon the thrombotic determinants discussed in the previous section, this subsection synthesizes the clinical impact of HC on specific CV and cardiometabolic risk factors.

Contraceptive counseling should incorporate baseline CV risk, method effectiveness and adherence needs, patient preferences, and changes in risk over time. Shared decision-making can be facilitated by discussing absolute rather than relative risks and emphasizing that pregnancy and the early postpartum period are associated with a substantially increased risk of VTE [44]. To support clinical counseling and risk communication, absolute VTE rates across different physiological states and contraceptive methods are compared in Table 3.

For women with high-risk conditions (e.g., prior VTE, known thrombophilia, migraine with aura, severe or uncontrolled HTN, complex congenital heart disease (CCHD), and multiple major atherosclerotic risk factors), estrogen-containing CHCs are generally avoided, and progestin-only or long-acting reversible methods are usually preferred. Eligibility frameworks such as the U.S. Medical Eligibility Criteria for Contraceptive Use 2024 provide structured condition-specific recommendations that can be incorporated into CV practice and interdisciplinary care [48]. When CHCs are considered appropriate, clinicians should use the lowest effective estrogen dose, reassess BP and smoking status periodically, screen for new-onset migraine with aura, and review co-administered medications—including NSAIDs—that may transiently increase the risk of thrombosis [8].

**Figure 1:**
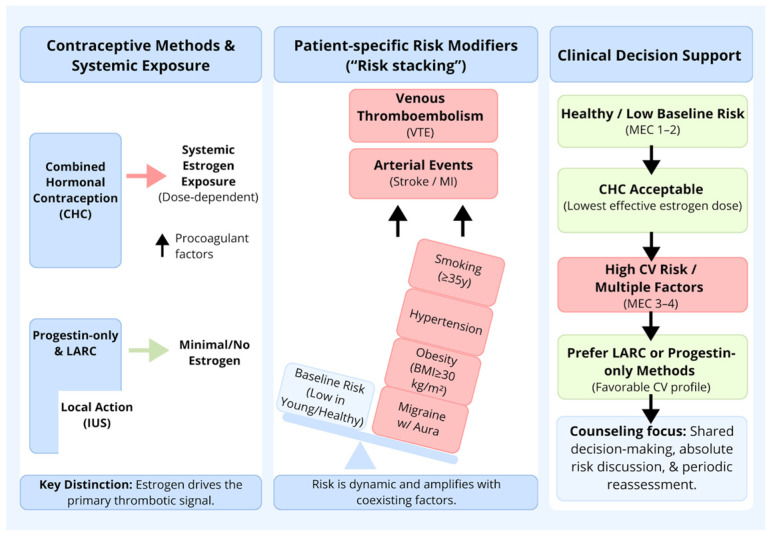
Visual abstract and clinical decision algorithm for cardiovascular risk assessment regarding hormonal contraception. Abbreviations: CHC—combined hormonal contraception, CV—cardiovascular, IUS—intrauterine system, LARC—long-acting reversible contraception, MEC—Medical Eligibility Criteria, MI—myocardial infarction, and VTE—venous thromboembolism.

We have synthesized the core concepts of exposure, risk assessment, and guideline-based decision-making into a visual algorithm (Figure 1).

### 2.6. Drug Interactions and Concomitant Medications

Drug interactions are clinically important in regard to HC because they can reduce contraceptive efficacy, modify the risk of thrombosis, and change exposure to concomitant therapies; therefore, a structured medication review is essential for eligibility assessment and follow-ups [48]. The most clinically important interactions that reduce effectiveness involve strong hepatic enzyme induction, particularly rifampin and rifabutin, which can lower systemic exposure to oral contraceptive steroids and permit ovulation, so non-estrogen methods and LARC are generally preferred when rifamycins are required [13,48]. Similar concerns apply to enzyme-inducing antiseizure medications (ASMs), with clinical pharmacology data showing that carbamazepine reduces contraceptive steroid levels and is associated with more breakthrough bleeding and ovulation while taking low-dose COCs, supporting the need to avoid estrogen-containing pills as a sole strategy and prioritize non-oral long-acting options when feasible [48,75]. Herbal products may also be clinically relevant because St. John’s wort triggers drug-metabolizing enzymes involved in contraceptive steroid clearance and has been associated with reduced hormone exposure, increased breakthrough bleeding, and signs of ovulation [76,77]. In contrast, most non-rifamycin antibiotics have not demonstrated clinically meaningful reductions in contraceptive effectiveness in pharmacokinetic and clinical outcome studies, and the interaction concern is mainly limited to rifampin-class antibiotics [13,78]. HC can also affect other drugs, most notably by increasing clearance of lamotrigine and substantially lowering its plasma concentrations during COC use, potentially requiring monitoring and dose adjustment when starting or stopping estrogen-containing contraception [79]. In CV practice, bosentan, used for pulmonary arterial HTN, decreases systemic exposure to EE and norethisterone, potentially reducing the effectiveness of oral HC. Therefore, highly effective non-oral long-acting methods are generally preferred for patients receiving bosentan [80]. Some concomitant therapies may increase the risk of thrombosis without reducing contraceptive efficacy. In large observational studies, NSAID use has been associated with a greater absolute increase in VTE among women using higher-risk HC than among those using lower-risk methods or no HC, a finding that may be clinically relevant for women who require regular NSAID therapy [8]. Finally, emerging drug interactions should also be considered. These include incretin-based therapies, where tirzepatide may reduce the absorption of oral HCs during treatment initiation and dose escalation. In practice, patients using oral HC should be advised to switch to a non-oral method or add a barrier method for 4 weeks after initiation and for 4 weeks after each dose escalation with tirzepatide [14,48,81].

## 3. Conclusions and Future Directions

Our review highlights the fact that the CV safety of HC is determined by the interaction between patient-level risk factors and estrogen exposure rather than by contraceptive use alone. A key practical message is that we must distinguish the dominant risk domain, VTE vs. arterial events, as these pathways differ clinically and lead to different choices of method. The greatest clinical impact arises from risk stacking, where multiple coexisting risk factors can translate a modest relative risk into a meaningful increase in absolute risk, so eligibility assessment should be approached as a longitudinal process rather than a one-time decision. Periodic reassessment of BP, smoking status, and new neurologic symptoms suggestive of migraine with aura, together with review of newly diagnosed comorbidities and concomitant medications, can improve safety over time. Our synthesis supports preferential use of non-estrogen options and LARC for women with high-risk profiles while emphasizing that absolute risks remain low in healthy young women and that method effectiveness, adherence, and patient preferences should remain central to shared decision-making. Risk communication should focus on absolute numbers and explicitly incorporate pregnancy-related CV risks, approaches that often shift the benefit–risk balance during counseling. Finally, future work should focus on developing risk prediction tools that integrate dynamic exposure and changing risk factors. Clinical safety enhancements will likely stem from the broader adoption of liver-neutral estrogens, such as E4 and natural estradiol, which have a reduced hemostatic footprint compared to traditional formulations. Generating more outcome data, particularly in regard to women with established CV disease, remains a priority in order to refine these personalized strategies and ensure long-term CV protection.

## Figures and Tables

**Table 1:** Common hormonal contraceptive methods and cardiovascular considerations.

Method	Typical Hormones	Dosing/Duration	Key Cardiovascular Considerations
COC (oral CHC)	EE (20–35 µg) + progestin (or E2/E4 + progestin)	Daily	It poses a higher risk of VTE relative to non-use. The risk varies by estrogen dose and progestin type. Generally, it should be avoided by individuals with migraines with aura, those with uncontrolled HTN, smokers ≥ 35 years, and those with a known risk of thrombophilia/at high risk of VTE.
Vaginal ring (CHC)	Etonogestrel + EE	3 weeks in/1 week out	There is systemic CHC exposure. The risk of VTE is greater relative to non-use in observational data. Patient risk factors must be considered (CHC eligibility criteria should be applied).
Transdermal patch (CHC)	Norelgestromin + EE	Weekly × 3/1 week off	PK studies suggest there is higher EE exposure than many COCs. Observational data suggest it poses an increased risk of thrombosis. High-risk patients should avoid use. Effectiveness may be reduced in individuals with greater body weight. Patch placement: Abdominal application is associated with ~20% lower NGMN/EE absorption than buttock/arm/torso placement, although mean levels remain within reference ranges.
POP (progestin-only pill)	Desogestrel, drospirenone, norethindrone, etc.	Daily	It is preferable when estrogen is contraindicated. Irregular bleeding is common. Risk of thrombosis is generally lower than that posed by CHC.
DMPA injection	Medroxyprogesterone acetate	Every 12–13 weeks	It may have adverse metabolic effects (regarding weight and lipids). Evidence on VTE is mixed, with limited data suggesting higher VTE odds mainly for injectable progestins. Individualization should be practiced for high-risk patients.
Etonogestrel implant	Etonogestrel	Up to 3 years	It is highly effective. There is a generally favorable thrombotic profile. Irregular bleeding can occur.
LNG-IUS (52 mg)	Levonorgestrel	Up to 8 years (evidence)	It has a predominantly local effect. There is minimal systemic hormone involvement. It has a favorable CV safety profile.

Abbreviations: CHC—combined hormonal contraception, COCs—combined oral contraceptives, CV—cardiovascular, DMPA—depot medroxyprogesterone acetate, E2—estradiol, E4—estetrol, EE—ethinyl estradiol, HTN—hypertension, LNG-IUS—levonorgestrel-releasing intrauterine system, NGMN—norelgestromin, PK—pharmacokinetics, POP—progestin-only pill, and VTE—venous thromboembolism.

**Table 2:** Impact of individual risk factors on cardiovascular profiles during hormonal contraception.

Risk Factor	Key Mechanism (Relevant to HC)	Predominant Cardiovascular Events	Practical Implications for Method Selection
Smoking (esp. age ≥ 35)	Endothelial dysfunction, platelet activation, and estrogen-related prothrombotic synergy	MI, ischemic stroke, and VTE	Smokers aged ≥35 years should avoid CHCs; CHCs are MEC 3 with <15 cigarettes/day and MEC 4 with ≥15 cigarettes/day. LNG-IUS, Cu-IUDs, implants, or POPs are preferable. Smoking cessation counseling should be offered [7,48,66].
Obesity (BMI ≥ 30 kg/m^2^)	Low-grade inflammation, insulin resistance, exacerbated coagulation factors, and impaired fibrinolysis	Mainly VTE—arterial risk rises with risk clustering	Obesity alone is MEC 2 for CHCs, but the risk of VTE increases further with additional risk factors. Consider LARC or POC when there is risk stacking [18,48].
Age (esp. ≥40)	Higher baseline vascular risk and more frequent comorbidities	Mainly arterial events (stroke/MI)	Age alone is not a contraindication, but risk is increased by comorbidity. Reassess eligibility over time and consider LARC or POC when additional risk factors coexist [48,67].
Hypertension	Endothelial dysfunction, increased shear stress, and estrogen-related BP and thrombotic effects	Mainly stroke/MI	Avoid prescribing CHCs to individuals with HTN whenever possible. CHCs are MEC 3 in individuals with controlled HTN or a BP of 140–159/90–99 mmHg and MEC 4 in individuals with a BP ≥ 160/100 mmHg or vascular disease. Preferably, select LARC or POC, and optimize BP control [48,68].
Diabetes mellitus	Endothelial dysfunction, inflammation, platelet hyperreactivity, and accelerated atherosclerosis	Mainly arterial events in long-standing or complicated disease	In uncomplicated diabetes, CHCs are usually MEC 2. In the presence of nephropathy, retinopathy, neuropathy, vascular disease, or a duration > 20 years, avoid estrogen when possible and select LARC or POC instead [48,69].
Migraine with aura	Altered cerebrovascular reactivity and an estrogen-related increase in stroke risk	Ischemic stroke	CHCs are MEC 4 and should be avoided. Preferably, select LNG-IUS, Cu-IUDs, implants, or POPs instead; reassess if aura develops during use [48,70].

Abbreviations: BMI—body mass index, BP—blood pressure, CHC—combined hormonal contraception, Cu-IUD—copper intrauterine device, HC—hormonal contraception, HTN—hypertension, IUD—intrauterine device, LARC—long-acting reversible contraception, LNG-IUS—levonorgestrel-releasing intrauterine system, MEC—Medical Eligibility Criteria, MI—myocardial infarction, POP—progestin-only pill, POC—progestin-only contraceptive, and VTE—venous thromboembolism.

**Table 3:** Risk of venous thromboembolism across different physiological and contraceptive states.

Exposure/Method	Risk of VTE	Unit	Risk Type	Clinical Relevance/Comment	References
No use of HC	3.01	per 10,000 woman-years	Absolute	Reference category for non-pregnant women.	[37]
Current use of oral HC (overall)	6.29	per 10,000 woman-years	Absolute	Approximately 2-fold higher risk than in non-users.	[37]
COCs with levonorgestrel	5.47	per 10,000 woman-years	Absolute	Lower risk than desogestrel-, gestodene-, or drospirenone- containing COCs.	[37]
Transdermal patch	9.7	per 10,000 exposure years	Absolute	Higher risk than in non-users.	[41]
Vaginal ring	7.8	per 10,000 exposure years	Absolute	Higher risk than in non-users.	[41]
Subcutaneous implant	Low risk; slight increase possible	–	Qualitative	Estimates are less precise, but absolute risk appears low.	[41]
Levonorgestrel IUS	No increase	–	Qualitative	Useful option when estrogen is contraindicated.	[41]
Progestin-only contraception overall	Generally, no significant increase	–	Qualitative	Clinically important alternative to combined hormonal contraception.	[11,40]
Pregnancy (all trimesters)	10–14	per 10,000 deliveries	Absolute	Overall pregnancy-associated VTE burden.	[74]
Antepartum period	6.5	per 10,000 person-years	Absolute	Risk is lower than that postpartum but higher than the non-pregnant baseline.	[74]
Postpartum period (first 12 weeks)	22.9	per 10,000 person years	Absolute	Highest absolute incidence among physiological states.	[74]
Relative daily risk of VTE vs. non-pregnant similarly aged women	3–10x higher antepartum and 12–35x higher postpartum	–	Relative	Magnitude of increased risk relative to age-matched non-pregnant women.	[74]

Abbreviations: VTE—venous thromboembolism, HC—hormonal contraception, COCs—combined oral contraceptives, and IUS—intrauterine system.

## Data Availability

No new data were created or analyzed in this study.
